# Editorial: Biogenic Amines and Neuromodulation of Animal Behavior

**DOI:** 10.3389/fnsys.2018.00031

**Published:** 2018-07-13

**Authors:** Irina T. Sinakevitch, Gabriella H. Wolff, Hans-Joachim Pflüger, Brian H. Smith

**Affiliations:** ^1^School of Life Sciences, Arizona State University, Tempe, AZ, United States; ^2^Department of Biology, University of Washington, Seattle, WA, United States; ^3^Department of Biology, Neurobiology, Freie Universiterlin, Berlin, Germany

**Keywords:** biogenic amine receptors, tyramine, octopamine, dopamine, serotonin

Neuropeptides and biogenic amines are important modulators in all nervous systems. Rather than having a specific “point-to-point” function, which is characteristic for classical neurotransmitters released from a presynaptic neuron onto a clearly defined postsynaptic neuron and usually associated with fast transmission in the millisecond range mediated by ionotropic receptor molecules, biogenic amines often act as neuromodulators having long lasting actions up to the second range always mediated by metabotropic receptor molecules (G-proteins) and different cellular signaling pathways. However, a particular chemical substance can either act as a fast neurotransmitter via ionotropic postsynaptic receptors or as a slow but long lasting neuromodulator via metabotropic postsynaptic receptor molecules. Therefore, substances like dopamine, serotonin or even tyramine and octopamine can act as fast neurotransmitters or slow neuromodulators. As such biogenic amines significantly change the efficacy of pre- to post-synaptic connections by affecting the cellular and biochemical properties of neurons. In this respect, neuromodulators are chemical substrate underlying plasticity in all nervous systems. Their range of action goes beyond affecting only the nervous system. Neuromodulators orchestrate a plethora of neuronal and physiological processes that together may serve a particular behavioral context or a specific physiological condition, and reciprocal interactions between the nervous system and metabolic or physiological states in non-nervous tissues are widely accepted as a new research focus. Neuropeptides like biogenic amines are released either in neuropiles of the central nervous system or in the peripheral nervous system.

Total nineteen articles (3 reviews, one perspective, and 15 originals) of this particular issue cover a broad range of topics related to biogenic amines. The first review by Ichinose et al. discusses the role of spontaneously firing dopaminergic neurons in the fruit fly brain and how they reflect the behavioral/internal state of the animal. Dopamine can have both roles, a fast neurotransmitter, a slow neuromodulator, depending on the receptor types of the postsynaptic neurons. Genetic manipulation of the activity of dopamine neurons resulted in changes to the behavioral state of the fly. Behaviors affected were sleep, sexual drive, hunger, and learning and memory. The second review article by Vonhoff and Keshishian discusses how during the development of neuromuscular connections in the fruit fly an interaction between the glutamatergic type I terminals of motoneurons and octopaminergic type II terminals of neuromodulatory neurons may be significant. Also, the growth cones of motoneurons respond to signals from partners via low-frequency calcium waves that may be crucial for regulating temporary and final connections. In the third review, Zhukovskaya and Polyanovsky studied the effects of various amines such as octopamine, tyramine, dopamine, and serotonin on olfactory and gustatory receptor neurons in insect antennae. Many amines are systemically released into haemolymph, an open circulatory system in insects supplies different body compartments, the dorsal or ventral cavity, the muscles, the central nervous system, etc. The authors suggest that the antenna may be a partially autonomous haemolymph compartment separated from other body parts.

An important aspect of chemical signaling is that all transmitters, whether they be classical transmitters, neuromodulators or hormones, can only act through their respective receptor molecules. Depending on which receptor type is activated, different signaling cascades can be triggered. In this issue, a number of articles are devoted to receptors:

Blenau et al. report on a fifth serotonin receptor in fruit flies - Dm5-HT2B - in addition to the three Dm5-HT1a, Dm5-HT1B, and Dm5-HT7 coupled to the cAMP signaling cascades and Dm5-HT2A leading to Ca2+ signaling through ITP. This fifth receptor is involved in controlling heartbeat and immune system function, and it can be antagonized by metoclopramide and mianserin.

Bridging to vertebrates, Der-Ghazarian et al. describe in mice a 5-HT1B receptor agonist—CP94253—which affects spontaneous and cocaine-induced locomotion and conditioned place preference. Aminergic receptors play key role in the development of addiction, and this study provides evidence that 5-HT1BR agonists may be used for anti-cocaine medications.

Aranda et al. report that in fruit flies an interesting mixed G-protein coupled receptor—DopEcR—which binds both dopamine and ecdysone and mediates ethanol-induced courtship sensitization. DopEcR immunoreactivity was observed in the mushroom body calyx and lobes, and in mutant DopEcR males, the sensitization phenotype was fully rescued by restoring DopEcR expression in mushroom body αβ and γ neurons.

The distribution of immunoreactivity for tyramine and one of its receptor molecules—AmTyr1—is described by Sinakevitch et al. for the honey bee brain and particular emphasis is placed on neuropils associated with olfactory learning and memory. They focus on two Ventral Unpaired Median neurons of the gnathal (suboesophageal) ganglion whose axons ascend to the brain and innervate the antennal lobe and mushroom body calyx. Interestingly, AmTyr1 expression was found in the presynaptic sites of olfactory receptor neurons and of uniglomerular projection neurons, most likely to exert inhibitory control of neurotransmitter release.

There is accumulating evidence that tyramine and octopamine exert opposite actions in insects. Ryglewski et al. in their study on fruit fly flight behavior, examine the role of tyramine and an enzyme for tyramine catabolism—dehydrogenase/reductase Naz (Nazgul). Naz is found in a particular group of glial cells that are located along the motor neuropil border and with extensions into the flight motor neuropil. If this enzyme is knocked down by RNAi, flight durations are reduced, which is typical for blocked octopamine and high tyramine levels. This article also discusses interesting pathways of tyramine signaling.

Biogenic amines are also involved in orchestrating responses associated with metabolic processes such as starvation. When starved tyrosine-ß-hydroxylase mutant fruit flies, which cannot synthesize octopamine, possess higher levels of glucose in their haemolymph than controls, as shown in the study by Damrau et al.. The article also reviews on the different receptor types of tyramine and octopamine that may be involved in energy homeostasis.

Most neurons that release octopamine belong to the class of dorsal unpaired median neurons, and their electrical properties have been extensively studied in cockroaches by Lapied et al.. They show that pacemaker activity of these neurons is facilitated by a tetrodotoxin-sensitive-low-voltage-activated channel permeable to sodium and calcium and regulated by a cAMP/PKA-cascade. Phospho-DARPP-32 strongly decreased this current and involved in regulating sodium/calcium-currents and contributing to pacemaker activity.

Amine malfunctions are often the causes of severe pathologies, such as Parkinson's disease. Niens et al. show that in the fruit fly, imbalances between dopamine and serotonin can be modeled. Like in rodents, a lack of dopamine leads to increased levels of 5-HT and arborizations in specific brain neuropils. Conversely, increased dopamine levels lead to the reduced connectivity of 5-HT neurons. This suggests that in Parkinson's disease, both dopamine and 5-HT play an important role.

Dopamine signaling is essential for mediating reinforcing properties of unconditioned stimuli during associative learning. Tedjakumala et al. characterize dopaminergic neurons in the honeybee brain by immunoreactivity distribution of the dopamine precursor enzyme, tyrosine-hydroxylase. They also describe new clusters of dopaminergic neurons.

In many social insects, like ants and bees, biogenic amines play functional roles in the control of sociality. How biogenic amines and their receptors in ancestral, solitary species have been co-opted during evolution to control behaviors in socially complex species is addressed by Kamhi et al..

Li et al. studied fat deposition or starvation resistence using flies defective in the expression of receptors for octopamine and tyramine. Their tissue-specific RNAi experiments revealed a very complex interorgan communication leading to the different metabolic phenotypes in octopamine- and tyramine-deficient fruit flies.

Sitaraman et al. described discrete neuronal circuits that mediate aversive reinforcement, escape latencies, and memory levels after place learning in the presence and absence of unexpected aversive events. The results show that two features of learned helplessness depend on the same modulatory system as aversive reinforcement. Moreover, aversive reinforcement and escape latency changes depend on local neural circuit modulation, while memory enhancement requires modulation of multiple behavioral control circuits.

Stocker et al. compared the axon terminals of octopaminergic efferent dorsal and ventral unpaired median neurons in either desert locusts or fruit flies across skeletal muscles, revealing many similarities. These type II terminals are immunopositive for both tyramine and octopamine and, in contrast to the type I terminals, which possess clear synaptic vesicles, they only consist of dense core vesicles. They discovered that starvation changes the morphology of the neuromuscular branches in a time-dependent manner. Besides, the authors provide evidence that the release of octopamine from dendritic and/or axonal type II terminals uses similar synaptic machinery to glutamate release from type I terminals of excitatory motor neurons.

Scheiner et al. investigated the role of the fat body in modulating gustatory responsiveness through tyramine signaling in different behavioral castes of honeybees. Their work suggests that differential tyramine signaling in the fat body has an essential role in the plasticity of division of labor through changing gustatory responsiveness.

Sun et al. studied startle-induced locomotion and the activity of specific clusters of dopaminergic neurons afferent to the mushroom bodies. Their study contributes to an emerging picture of the brain circuits modulating locomotor reactivity in fruit flies that appear to both overlap and differ from those mediating associative learning and memory, sleep/wake state and stress-induced hyperactivity.

Buckemüller et al. investigated alterations of haemolymph glucose concentration, survival, and feeding behaviors after starvation and examined the impact of octopamine on these processes in pharmacological experiments. Their experiments demonstrated that octopamine in honey bees acts similarly to adrenalin and noradrenalin in mammals in regulating an animal's counter-regulatory response.

## Author contributions

All authors listed have made a substantial, direct and intellectual contribution to the work, and approved it for publication.

### Conflict of interest statement

The authors declare that the research was conducted in the absence of any commercial or financial relationships that could be construed as a potential conflict of interest.

